# Vitamin D to Prevent Lung Injury Following Esophagectomy—A Randomized, Placebo-Controlled Trial*

**DOI:** 10.1097/CCM.0000000000003405

**Published:** 2018-11-16

**Authors:** Dhruv Parekh, Rachel C. A. Dancer, Aaron Scott, Vijay K. D’Souza, Phillip A. Howells, Rahul Y. Mahida, Jonathan C. Y. Tang, Mark S. Cooper, William D. Fraser, LamChin Tan, Fang Gao, Adrian R. Martineau, Olga Tucker, Gavin D. Perkins, David R. Thickett

**Affiliations:** 1 Birmingham Acute Care Research Group, Institute of Inflammation and Aging, University of Birmingham, Birmingham, United Kingdom.; 2 Queen Elizabeth Hospital University Hospitals, Birmingham NHS Foundation Trust, Birmingham, United Kingdom.; 3 Warwick Clinical Trials Unit, Warwick Medical School, University of Warwick, Coventry, United Kingdom.; 4 Academic Department of Anaesthesia, Critical Care, Resuscitation and Pain, Heartlands Hospital, University Hospitals Birmingham NHS Foundation Trust, Birmingham, United Kingdom.; 5 Norwich Medical School, University of East Anglia, Norwich, United Kingdom.; 6 Discipline of Medicine, Concord Clinical School, University of Sydney, NSW, Australia.; 7 University Hospitals Coventry and Warwickshire NHS Trust, Coventry, United Kingdom.; 8 Blizard Institute, Queen Mary University of London, London, United Kingdom.

**Keywords:** acute respiratory distress syndrome, cholecalciferol, esophagectomy, vitamin D

## Abstract

**Objectives:**

Observational studies suggest an association between vitamin D deficiency and adverse outcomes of critical illness and identify it as a potential risk factor for the development of lung injury. To determine whether preoperative administration of oral high-dose cholecalciferol ameliorates early acute lung injury postoperatively in adults undergoing elective esophagectomy.

**Design:**

A double-blind, randomized, placebo-controlled trial.

**Setting:**

Three large U.K. university hospitals.

**Patients:**

Seventy-nine adult patients undergoing elective esophagectomy were randomized.

**Interventions:**

A single oral preoperative (3–14 d) dose of 7.5 mg (300,000 IU; 15 mL) cholecalciferol or matched placebo.

**Measurements and Main Results:**

Primary outcome was change in extravascular lung water index at the end of esophagectomy. Secondary outcomes included Pao_2_:Fio_2_ ratio, development of lung injury, ventilator and organ-failure free days, 28 and 90 day survival, safety of cholecalciferol supplementation, plasma vitamin D status (25(OH)D, 1,25(OH)_2_D, and vitamin D-binding protein), pulmonary vascular permeability index, and extravascular lung water index day 1 postoperatively. An exploratory study measured biomarkers of alveolar-capillary inflammation and injury. Forty patients were randomized to cholecalciferol and 39 to placebo. There was no significant change in extravascular lung water index at the end of the operation between treatment groups (placebo median 1.0 [interquartile range, 0.4–1.8] vs cholecalciferol median 0.4 mL/kg [interquartile range, 0.4–1.2 mL/kg]; *p* = 0.059). Median pulmonary vascular permeability index values were significantly lower in the cholecalciferol treatment group (placebo 0.4 [interquartile range, 0–0.7] vs cholecalciferol 0.1 [interquartile range, –0.15 to –0.35]; *p* = 0.027). Cholecalciferol treatment effectively increased 25(OH)D concentrations, but surgery resulted in a decrease in 25(OH)D concentrations at day 3 in both arms. There was no difference in clinical outcomes.

**Conclusions:**

High-dose preoperative treatment with oral cholecalciferol was effective at increasing 25(OH)D concentrations and reduced changes in postoperative pulmonary vascular permeability index, but not extravascular lung water index.

## Abstract 2

Supplemental Digital Content is available in the text.

Prevention of the acute respiratory distress syndrome (ARDS) has become a focus of research in recent years, with the development of prediction scores (1), prevention trials (2, 3) and a growing evidence of other potential biological therapies to prevent ARDS (4).

ARDS is common after esophagectomy with studies reporting a frequency between 13% and 30% (2, 5–7). During open surgery, access to the esophagus is attained by deflation of one lung and maintenance of one-lung ventilation (OLV) exposing the ventilated lung to volutrauma, hyperoxia, and barotrauma (8, 9). Concurrently the deflated lung is exposed to ischemia-reperfusion injury (10). The pathophysiologic changes of lung injury post esophagectomy are similar but less exaggerated than in ARDS patients (11–13). This high-risk population has been validated for assessing potential therapies to assess biological efficacy and prevent ARDS (2).

Vitamin D (VD) deficiency is common (14). VD has important functions besides bone, and calcium homeostasis and its immunomodulatory actions may play a role in the pathogenesis of ARDS (15). Studies report a high prevalence of VD deficiency (VDD) in the critically ill (16) with an association with increased rates of infection (17, 18) acute respiratory failure (19), acute kidney failure (20), sepsis (18, 21), and mortality in some (22), but not all studies (23, 24).

We have previously reported severe VDD in a cohort of ARDS patients (25). VDD (plasma 25(OH)D < 50 nmol/L) was ubiquitous in ARDS patients and present in the vast majority of patients at risk of developing ARDS following esophagectomy. Experimental studies suggest a protective effect of VD in the lung. In a murine model of intratracheal lipopolysaccharide challenge, dietary-induced VDD resulted in exaggerated alveolar inflammation, epithelial damage, and hypoxia which were abrogated by cholecalciferol treatment (21). In vitro, 25(OH)D has trophic effects on primary human alveolar type II cells affecting over 600 genes (25). We conducted a phase 2 randomized, placebo-controlled trial to test our hypothesis that high-dose cholecalciferol treatment preoperatively reduces markers of alveolar epithelial lung injury seen post esophagectomy, a high-risk population of developing ARDS. Results of these studies have been previously reported in the form of an abstract (26).

## MATERIALS AND METHODS

### Study Design

A randomized, double-blind placebo-controlled trial, recruited patients from October 3, 2012, to January 26, 2015, at three hospitals in the United Kingdom. Ethics approval was obtained from South Birmingham Research Ethics Committee (REC 12/WM/0092). Trial registration identification ISRCTN27673620 and EudraCT 2012-000332-25. The study protocol has been previously published (27). Detailed methods can be found in the **online supplemental material** (Supplemental Digital Content 1, http://links.lww.com/CCM/D967).

### Eligibility Criteria

Patients undergoing a planned thoracic esophagectomy and 18 years old or older if male, aged 55 or more than 2 years since menopause if female and were able to give written informed consent. Exclusion criteria detailed in the online supplemental material (Supplemental Digital Content 1, http://links.lww.com/CCM/D967).

### Drug Randomization and Masking

The trial drug manufacturer, Novalabs (Leicester, United Kingdom), produced computer-generated randomization sequence using a block size of 10 with equal allocation between active and placebo groups. Pharmacy, research staff, clinical teams, and patients were masked to randomization and treatment allocation.

### Drug Administration

Participants received either a single dose of drug (oral cholecalciferol oily solution Vigantol (Novalabs), 300,000 IU [7.5 mg; 15 mL]) or matched placebo (Miglyol 812 oil, the vehicle for cholecalciferol in Vigantol) 3–14 days prior to planned esophagectomy.

### Primary Outcome

Primary outcome was change in extravascular lung water index (EVLWI) measured by Pulse Contour Continuous Cardiac Output (PiCCO_2_) thermodilution catheter (Pulsion Medical Systems, Feldkirchen, Germany) at the end of the esophagectomy (measured within 1 hr postoperatively). PiCCO_2_ EVLWI has been shown to be a marker for developing lung injury (28) and mortality in ARDS (29, 30) and has been used as the primary outcome in clinical trials in ARDS (31, 32) as well as post thoracotomy (33).

### Secondary Outcomes

Prespecified trial secondary outcomes were Pao_2_:Fio_2_ ratio, development of lung injury in the first 28 days as defined by the Berlin Criteria, ventilator and organ failure free days, 28 and 90 day survival and safety (e.g., hypercalcemia), and tolerability of cholecalciferol supplementation. Plasma VD status (25(OH)D, 1,25(OH)_2_D, and vitamin D binding protein DBP) predrug dose, preoperatively, postoperatively, and day 3 as well as EVLWI day 1 postoperatively (measured at 9 am on day 1). PiCCO_2_ derived pulmonary vascular permeability index (PVPI) was added as an outcome measure prior to the conduct of the study as recent data suggest it was a better measure of alveolar-capillary permeability to differentiate nonhydrostatic edema than EVLWI (34).

### Exploratory Outcomes

Details are available in the online supplemental material (Supplemental Digital Content 1, http://links.lww.com/CCM/D967).

### Lung Water Measurements

EVLWI and PVPI were measured by thermodilution (PiCCO_2_) as previously described (35, 36). Specified time points for measurement were immediately preoperatively, within 1 hour postoperatively and day 1 postoperatively (8–9 am). Detailed methods are provided in the online supplemental material (Supplemental Digital Content 1, http://links.lww.com/CCM/D967) and published trial protocol (27).

### Perioperative Care

Patients underwent a two-stage transthoracic esophagectomy which included a laparoscopic abdominal stage followed by an open thoracotomy or minimally invasive technique with thoracoscopy. All approaches required OLV. Anesthesia was performed dependent on the anesthetists preferred practice. Teams were briefed to adopt a lower tidal volume and fluid-conservative hemodynamic management approach.

### Sample Size

The trial was powered to detect a change of 20% in EVLWI with a power of 80% requiring 34 patients (68 in total) in each arm to reach the primary endpoint (two-tailed *p* = 0.05). An additional six patients were added to allow for dropouts, such as open/close cases, unexpected deaths, and other difficulties with data collection. Detailed information about the sample size estimate is provided in the trial protocol (27).

### Statistical Analysis

Data were analyzed using Graphpad PRISM 6 software package (Graphpad, San Diego, CA). All analysis was based on Intention to treat. Continuous data were assessed for normality using the Shapiro-Wilks test and the appropriate parametric or nonparametric test applied. Statistical significance was predefined as two-tailed *p* value of less than 0.05. To assess the significance of differences between two sets of continuous data the unpaired *t* test was used for parametric data, whereas the Mann-Whitney *U* test was used for nonparametric data, except paired data which were assessed using paired *t* test (parametric) or Wilcoxon signed rank test (nonparametric). Categorical data were assessed using Fisher exact test and chi-square test for larger samples.

## RESULTS

### Enrollment and Patient Characteristics

**Figure 1.**
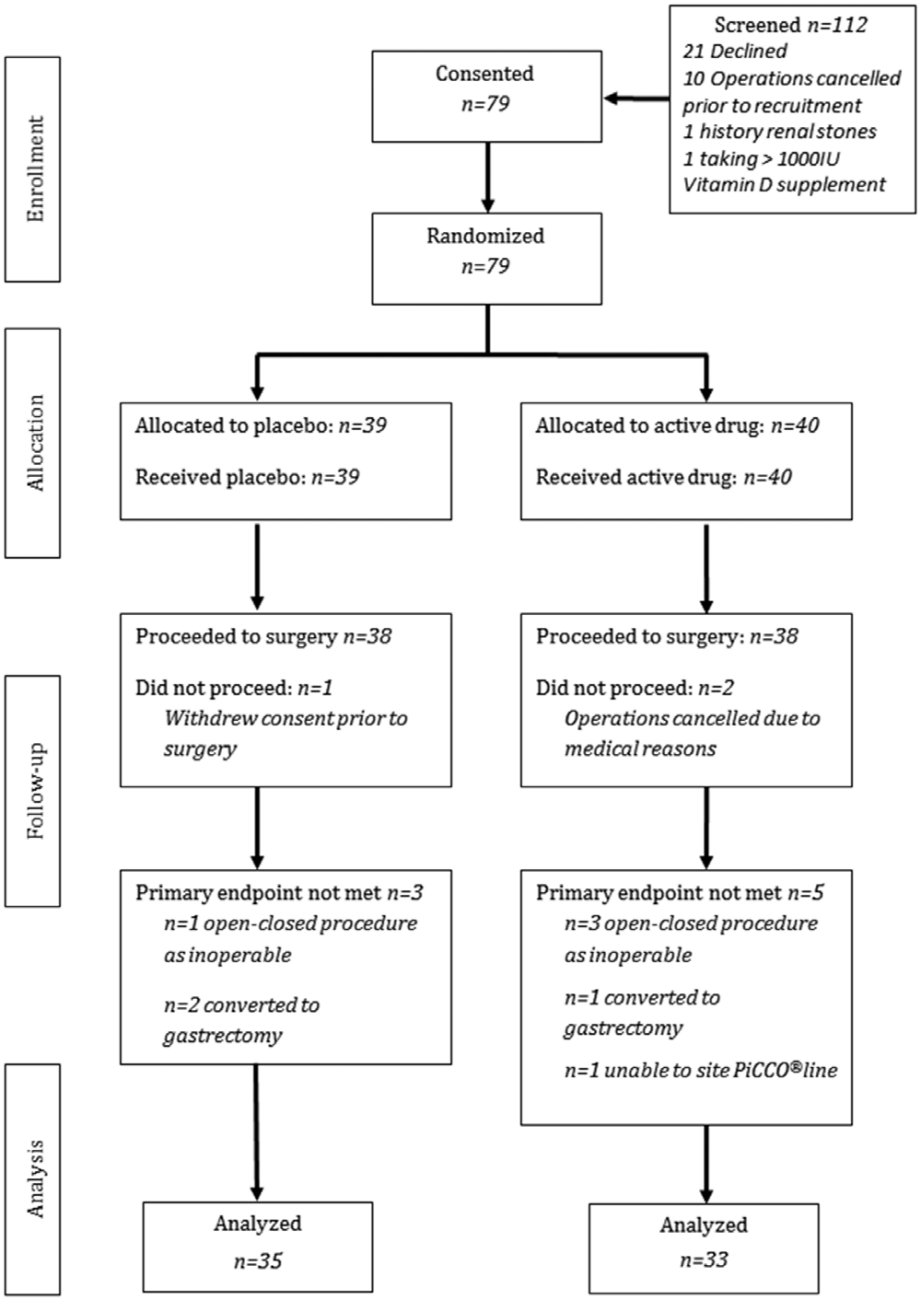
Patient Consolidated Standard of Reporting Trials flow diagram. IU = international unit, PiCCO = Pulse Contour Continuous Cardiac Output.

A total of 79 patients were enrolled in the study (Consolidated Standard of Reporting Trials) (**Fig. 1**). Eleven patients (14%) did not receive OLV, or it was not possible to measure the primary endpoint (EVLWI at the end of surgery). Thirty-five patients in the placebo arm and 33 in the cholecalciferol arm (*n* = 68; 86%) went on to complete esophagectomy and primary outcome measurement. All patients (*n* = 76; 96%) that proceeded to surgery were included in the analysis of efficacy and safety of cholecalciferol supplementation.

Patient baseline characteristics were well matched (**Table 1**). All procedures were performed for malignancy, and the predominant cell type was adenocarcinoma. Groups were well matched with respect to operative and anesthetic interventions (**Table 2**).

**TABLE 1.**
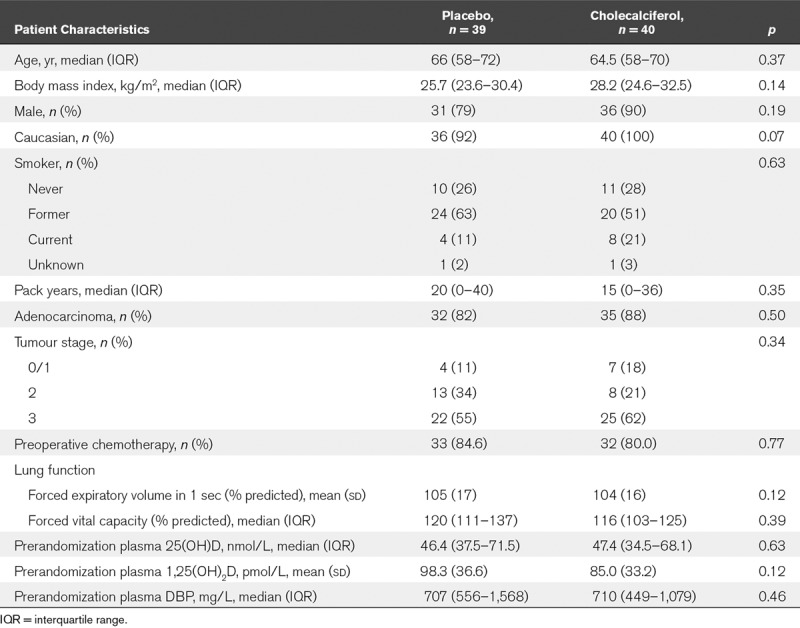
Patient Baseline Characteristics

**TABLE 2.**
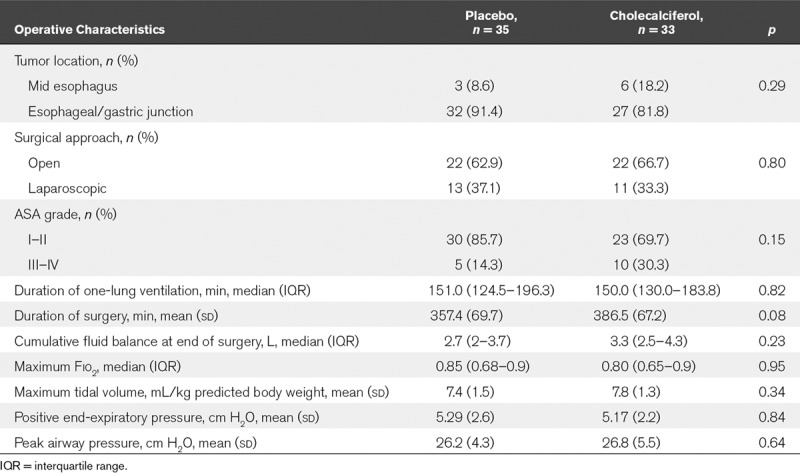
Anesthetic and Operative Characteristics

### Primary Outcome

**Figure 2.**
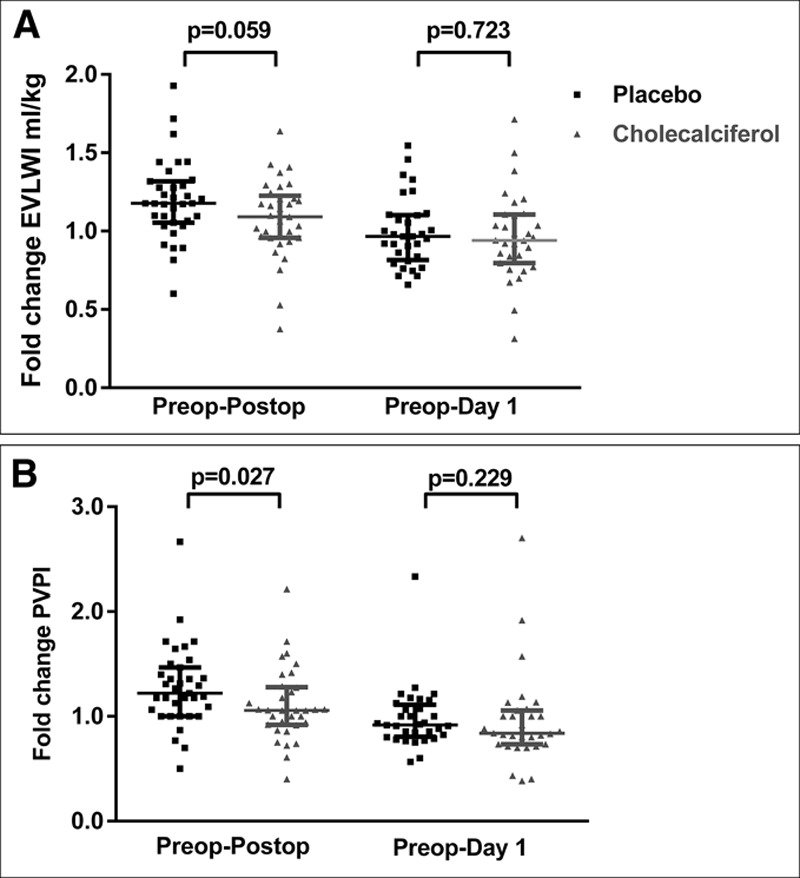
**A**, Scatter plot of fold change in extravascular lung water index (EVLWI). **B**, Scatter plot of fold change in pulmonary vascular permeability index (PVPI). Fold changes shown from pre- to postoperative and preoperative to day 1; *black lines* and *squares dots* placebo (*n* = 35); *gray lines* and *triangle dots* cholecalciferol treatment (*n* = 33); and data presented as medians and interquartile ranges. *p* values represent Mann-Whitney *U* test.

There was no difference in the a priori primary outcome of change in EVLWI measured within 1 hour postoperatively placebo median +1.0 (interquartile range [IQR], 0.4–1.8) versus cholecalciferol median +0.4 mL/kg (IQR, –0.4 to –1.2 mL/kg); *p* value equals to 0.059 (**Fig. 2*A***). There was no difference in absolute EVLWI between placebo and cholecalciferol arms postoperatively or postoperative day 1 time points (**Table E1**, Supplemental Digital Content 1, http://links.lww.com/CCM/D967). In a within-group analysis, EVLWI rose significantly preoperatively to postoperatively only in the placebo group (**Fig. E1**, Supplemental Digital Content 1, http://links.lww.com/CCM/D967).

### Secondary Outcomes

***Effects on Perioperative Changes in PVPI.*** The change in PVPI in placebo patients was median 0.4 (IQR, 0–0.7) versus cholecalciferol median 0.1 (IQR, –0.15 to –0.35); *p* value equals to 0.027 (**Fig. 2*B***). There was no difference seen in absolute values of PVPI preoperatively, postoperatively, and at day 1 between the groups (**Table E2**, Supplemental Digital Content 1, http://links.lww.com/CCM/D967). In a within-group analysis PVPI significantly increased pre- to postoperatively in patients who received placebo, but not VD (**Fig. E2**, Supplemental Digital Content 1, http://links.lww.com/CCM/D967).

***Safety of Cholecalciferol Supplementation.*** Trial medication was well tolerated with no episodes of hypercalcemia and no reported serious adverse events related to the trial medication (detailed in the online supplemental material and **Table E3**, Supplemental Digital Content 1, http://links.lww.com/CCM/D967).

***Efficacy of Cholecalciferol Supplementation Upon VD Status Perioperatively.*** There was no difference between groups in baseline 25(OH)D, 1,25(OH)_2_D, or DBP levels (Table 1). A single bolus dose of 300,000 IU of cholecalciferol resulted in significant increases in plasma 25(OH)D concentration (**Table 3**).

**TABLE 3.**
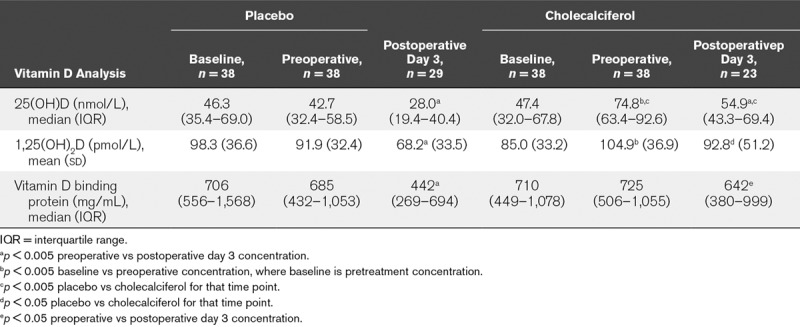
Perioperative Change in Vitamin D Status

On the day of operation 58% of patients who received placebo were deficient (25(OH)D concentrations < 50 nmol/L) compared with 5% of patients who had received the cholecalciferol single bolus dose (χ^2^ [1, *n* = 76] =22.36; *p* < 0.0001).

1,25(OH)_2_D concentrations increased significantly in patients randomized to receive cholecalciferol (Table 3) although this did not result in significantly different 1,25(OH)_2_D levels between groups. There was no significant change in plasma DBP concentrations.

Following the surgical insult concentrations of 25(OH)D and DBP decreased in both groups, but there were persistently higher 25(OH)D concentrations in the cholecalciferol treated arm day 3 postoperatively (Table 3). This meant that in the placebo group VDD was present in 58% on the day of operation and 90% by day 3. For the cholecalciferol supplemented patients, 5% were deficient at the time of operation increasing to 49% by day 3 postoperatively, demonstrating that critical illness induces VDD in this patient group for the first time.

***Clinical Outcomes.*** There was no significant difference seen in Pao_2_:Fio_2_ ratio between the groups. There was no difference in ARDS rates between placebo and cholecalciferol treatment arms (placebo 4 [11%] of 35 compared with cholecalciferol 4 [12%] of 33; odds ratio, 0.94; 95% CI, 0.21–4.09). There was no difference seen in ventilator-free and organ failure free days or survival (28 or 90 d) (detailed in the online supplemental material, Supplemental Digital Content 1, http://links.lww.com/CCM/D967; and **Table E4**, Supplemental Digital Content 1, http://links.lww.com/CCM/D967). No patients developed renal or cardiac failure in the first 7 days.

***Exploratory Outcomes.*** Details can be viewed in **Table E5** (Supplemental Digital Content 1, http://links.lww.com/CCM/D967).

***Exploratory Post Hoc Analysis of EVLWI and PVPI Measurements.*** Only 58 % of placebo-treated and 5% of cholecalciferol treated patients were VDD on the day of the operation. Therefore a post hoc exploratory-pooled analysis of all patients irrespective of treatment arm was performed to determine if there is a threshold effect above which the beneficial effects of 25(OH)D on EVLWI and PVPI are seen. VD sufficient patients (> 50 nmol/L) on the day of operation had significantly lower increases in EVLWI and PVPI compared with those who were deficient irrespective of treatment allocation (**Fig. E3**, Supplemental Digital Content 1, http://links.lww.com/CCM/D967).

## DISCUSSION

In this phase 2 biological efficacy study, we sought to determine if high-dose cholecalciferol replacement attenuates the increase in alveolar-capillary permeability and development of pulmonary edema after esophagectomy. The main finding was that 300,000 IU of oral cholecalciferol treatment pre-esophagectomy did not reduce the change in EVLWI postoperatively but did reduce the change in PVPI.

Vitamin D3, or cholecalciferol, is mainly formed in the skin after exposure to sunlight (ultraviolet B). Synthesized or dietary VD is hydroxylated in the liver by CYP27A1, CYP2R1, and CYP3A4 to 25-hydroxyvitamin D_3_ [25(OH)D] the major circulating form of VD which is widely accepted as the key measure of VD status (37). In humans, 25(OH)D is bound to its binding protein (DBP) and requires hydroxylation to the active 1,25-dihydroxyvitamin D_3_ [1,25(OH)_2_D] by the mitochondrial enzyme CYP27B1 (37) in order to activate the VD receptor (VDR) which mediates the biological actions of 1,25(OH)_2_D. Both CYP27B1 and VDR are expressed by cells of the innate and adaptive immune system as well as alveolar epithelial cells (38, 39).

Our hypothesis was based upon experimental data: alveolar epithelial cells possess the ability to convert circulating 25(OH)D to active 1,25(OH)_2_D and activate VDR responsive genes (39), suggesting organ-specific effects. Physiologically relevant doses of 25(OH)D stimulate wound repair, cellular proliferation, and reduce soluble Fas ligand induced cell death of human type 2 alveolar epithelial cells in vitro (25), suggesting that cholecalciferol has a direct protective role on the alveolar epithelium. There may also be a protective mechanism on the pulmonary endothelium as 1,25(OH)_2_D has been shown to decrease expression of intercellular adhesion molecule-1 and prevent neutrophil adhesion, migration and therefore potentially the initiation of lung injury (40). Furthermore, observational studies have found that VDD is common in patients with sepsis and ARDS and an increased risk of developing ARDS in patients undergoing esophagectomy (21, 25).

The changes in PVPI and EVLWI, we observed in this study were smaller than the observational studies led us to expect. This may reflect a change in baseline VD status in the population studied, which were not as deficient as the patient cohort that this study was powered on—median 25(OH)D 25.5 nmol/L (25) compared with 43.2 nmol/L in the current study, suggesting that the beneficial effects of replacement may be greater in those with more severe VDD. Additionally, improved surgical technique and anesthetic management may also explain the smaller postoperative increases in EVLWI and PVPI between the two studies that were 5 years apart.

High-dose cholecalciferol successfully increased 25(OH)D concentrations above sufficiency (50 nmol/L) in 94.7% of cases leading to a corresponding and sustained rise in 1,25(OH)_2_D level perioperatively. However, following surgical trauma induced by esophagectomy, both arms had significant falls in 25(OH)D day 3 postoperatively demonstrating that critical illness can rapidly induce a state of 25(OH)D deficiency. The placebo group also saw similar falls in circulating 1,25(OH)_2_D perioperatively but not in the patients who received cholecalciferol, indicating that patients in the placebo group have insufficient circulating 25(OH)D to maintain plasma concentrations of 1,25(OH)_2_D in the perioperative period.

It is unclear how critical illness promotes this rapid fall, but our data suggest that perioperative falls in circulating DBP, the major carrier protein produced by the liver may be important. Although this could be accounted for by hemodilution (41), the cause of loss of binding proteins in acute inflammation is still unknown and could also be related to interstitial extravasation from increased vascular permeability following inflammatory responses and decreased hepatic synthesis (42). In addition, enhanced induction of activation to 1,25(OH)_2_D or catabolic pathways such as the formation of 24-hydroxylated or three epimer forms of VD that have recently been identified may also play a role (43).

There is also evidence that VD may be a negative acute-phase reactant postelective knee arthroscopy (44) and in acute pancreatitis in which it recovers without supplementation (45). We believe this is an important area for future research as it is critical to inform dosing regimens for further studies into rapid VD replacement in critically ill patients. Patients may require repeat dosing rather than the large single doses so far investigated and serum 25(OH)D may be an unreliable marker of VD status after an acute inflammatory insult (46).

Importantly high-dose cholecalciferol therapy was well tolerated with no frequency of hypercalcemia. The frequency of ARDS in this cohort was 11.7%. Cholecalciferol treatment did not prevent the development of ARDS, and there was no difference in other clinical outcomes, although our study was not powered to detect differences in clinical outcomes.

This study has limitations. The changes observed in PVPI and EVLWI were much lower than previously observed possibly due to the current cohort being very much less VD deficient than previously with significant numbers of patients in the control arm not being VD deficient. The lower than expected PiCCO changes and low frequency of ARDS may have led to a type II error with no difference seen in the primary outcome. The timing of the dose was not uniform, but this did not impact the level of sufficiency in the VD group on the day of operation. Our primary outcome was a measure of potential in vivo efficacy of VD on the lung barrier, but measurements of PiCCO EVLWI have limitations of under- and over-estimation due to positive end-expiratory pressure, changes in pulmonary vascular occlusion, heterogeneous lung injury, and operator-dependency (30). Finally, recent studies suggest that higher doses than we used and alternative routes of administration may be more efficacious in critical illness (47) and sepsis (48), but none have investigated effects on lung injury. Our results also support the hypothesis of benefit in the more deficient cohort and the use of a follow-up maintenance dose which we did not use. Clearly, the optimum dose of VD, best route of administration, complex metabolic pathways, and multiple isoforms that influence its bioactivity need to be established in sepsis, ARDS, and critical illness before large-scale trials are undertaken.

## CONCLUSIONS

A single preoperative dose of cholecalciferol did not reduce EVLWI in the human esophagectomy model of lung injury. There was some effect on PVPI. Surgical insult results in a precipitous decrease in VD concentrations, which is an important consideration for future trial design to address clinical efficacy of VD therapy in ARDS and critical illness. It is clear that the ability to rapidly identify critically ill patients who are deficient in VD is a necessary precursor to trial enrollment of VD replacement in critically ill patients.

## ACKNOWLEDGMENTS

We thank all the research, preoperative nursing, anesthetic, and surgical teams at University Hospitals Birmingham NHS Foundation Trust, Heart of England NHS Foundation Trust, and University Hospital Coventry and Warwickshire NHS Trust. In particular, Dr. Simon Smart, Dr. Jeremy Marwick, Mr. John Whiting, Mr. Ewen Griffiths, and Professor Derek Alderson from the University Hospitals Birmingham NHS Foundation Trust; Mr. Raj Nijjar, Mr. Martin Richardson; and Dr. Ruth McKenzie from the Heart of England NHS Foundation Trust. We are extremely grateful to Dr. Keith Couper and Teresa Melody from the Academic Department of Anesthesia, Critical Care, Resuscitation and Pain Department, Heart of England NHS Foundation Trust for helping to facilitate patient recruitment and trial delivery, and Dr. Anita Pye for providing overall trial support and coordination. We are grateful to Mr. Rajnikant Mehta for his independent senior statistical review of the data analysis and interpretation.

## Supplementary Material
