# Supplementary material for: Vitamin D to Prevent Lung Injury Following Esophagectomy—A Randomized, Placebo-Controlled Trial*
Source: Crit Care Med. 2018 Nov 16;46(12):e1128–35. doi: 10.1097/CCM.0000000000003405 (PMC6250246; doi:10.1097/CCM.0000000000003405)
Supplement: Supplementary file 1 [file ccm-46-e1128-s001.docx]

**Online Supplementary Material**

**Vitamin D therapy to prevent lung injury following esophagectomy (VINDALOO) – a randomized placebo controlled trial.**

Dhruv Parekh^1-3*,^ Rachel CA Dancer^1,4*^, Aaron Scott^1^, Vijay D’Souza^1^, Phillip A Howells^1^, Rahul Mahida^1^, Jonathan CY Tang^5^ ,Mark S Cooper^6^, William D Fraser^5^, LamChin Tan^7^, Fang Gao^1,4^, Adrian R Martineau^8^, Tucker O^1,4^, Gavin D Perkins^3,4^, David R Thickett^1,2^

**METHODS**

***Exclusion Criteria***

Patients with a known intolerance of VD, pregnant or breast feeding, sarcoidosis, tuberculosis, lymphoma, hyperparathyroidism, or nephrolithiasis, baseline serum adjusted calcium >2.65mmol/L, undergoing hemodialysis, or had a known diagnosis of chronic obstructive pulmonary disease (COPD) with FEV1 less than 50% predicted or resting oxygen saturations less than 92% were ineligible.

Patients taking following concomitant medication were excluded – more than 1,000IU (25mcg)/day VD supplementation a month preceding or more than 10mcg/day in the 2 months preceding enrolment; cardiac glycoside, carbamazepine, phenobarbital, phenytoin, primidone, or long-term immunosuppressant therapy; thiazide diuretic at a dose higher than recommended in the British National Formulary or in combination with a calcium supplement.

***Drug Randomization and Masking***

The trial drug manufacturer, Novalabs (Leicester, UK), produced the computer generated randomization sequence using a block size of 10 with equal allocation between active and placebo groups. Drug (oral cholecalciferol oily solution Vigantol™, 300,000 IU (7.5 mg)) and matching placebo (Miglyol 812 oil, the vehicle for cholecalciferol in Vigantol™) were packaged according to the randomization sequence into numbered treatment boxes. Drug boxes were supplied to centers in blocks of 10 thus ensuring an equal allocation between active and placebo groups to balance any differences in case mix, pre-operative, operative and post-operative care between centers. Patients were randomized sequentially by allocating them to the next numbered treatment pack held at the center. Pharmacy, research staff, clinical teams, and patients were masked to randomization and treatment allocation. Active and placebo treatment packs and their contents were identical in appearance. Trial staff directly observed patients taking drug to ensure 100 % compliance.

The protocol allowed for emergency un-blinding in the event of significant concerns about patient safety. Subjects could withdraw from the trial or the trial treatment at any time without prejudice. If a subject withdrew from the trial treatment, then they were followed-up wherever possible and data collected as per protocol until the end of the trial.

***Perioperative Care***

Patients underwent a 2-stage transthoracic esophagectomy which included a laparoscopic abdominal stage followed by an open thoracotomy or minimally invasive technique with thoracoscopy. All approaches required one-lung ventilation. Induction of anesthesia was performed intravenously and dependent on the anesthetists preferred practice. Teams were briefed to adopt a lower tidal volume and fluid-conservative hemodynamic management approach.

***Lung Water and Plasma Measurements***

Detailed methods are provided in the published trial protocol (1). EVLWI and PVPI were measured by thermodilution (PiCCO_2_^®^; Pulsion Medical Systems, Feldkirchen, Germany) preoperatively (prior to knife-to-skin), at the end of the procedure (within 1 hour postoperatively), and the first postoperative day as previously described between 0800-0900 (2, 3). We ensured that the sequential measurements on an individual patient was only done by one member of the research staff and only 2 research fellows ever performed any of the readings to minimise variability and error. EVLWI was measured as an indirect assessment of the accumulation of fluid in the interstitum and alveolar space (4). PVPI reflects alveolar capillary permeability (5).

Blood was collected pre-drug dosing, preoperatively, at the end of the procedure, and the morning after surgery (8–10 am) and again at day 3 postoperatively. Samples were processed to obtain plasma and frozen at −80°C (6).

Total 25(OH) Vitamin D was measured by tandem mass spectrometry using appropriate

National Institute of Standards and Technology aligned material achieving certification from the vitamin D External Quality Assessment Scheme (DEQAS) (7). 1,25(OH)_2_ Vitamin D levels were measured by enzyme immunoassay (EIA) (immunodiagnostic systems Ltd, Bolden, UK) and DBP was measured by ELISA (immundiagnostik, Bensheim, Germany).

Inflammatory mediators (IL-6, IL-8, IL1-β, tumour necrosis factor [TNF]-α) were measured by Magnetic Luminex^®^ Performance Assays (R&D Systems, Abingdon, UK) Soluble receptors for advanced glycation endproducts [sRAGE] and TNF-R1 and R2 were measured by MILLIPLEX^®^ map assays (Millipore, Watford, UK) at a central laboratory according to manufacturer’s instructions.

**RESULTS**

***Patient Recruitment***

All patients (n=79) received their allocated treatment. One patient (2.6%) in the placebo arm withdrew consent prior to surgery and 2 (5%) in the cholecalciferol arm did not proceed to surgery post-randomization for medical reasons. Intra-operatively one patient (2.6%) in the placebo arm and three (7.9%) in the cholecalciferol arm had inoperable malignancy and two patients (5.3%) in the placebo arm and 1 (2.6%) in the cholecalciferol arm were converted to extended total gastrectomy. We were unable to place a PiCCO^®^ catheter for one patient (2.6%) in the cholecalciferol arm due to anatomical reasons.

***Extravascular lung water index (EVLWI)***

| EVLWI (ml/kg)  *median (IQR)* | Placebo  *n=35* | Cholecalciferol  *n=33* | p-value |
| --- | --- | --- | --- |
| Preoperative | 6.3 (5.3- 7.7) | 6.2 (5.3 -7.7) | 0.819 |
| Postoperative | 7.1 (6.0 – 9.5) | 6.9 (5.8 – 7.7) | 0.269 |
| Day 1 | 5.9 (5.0 – 7.7) | 5.7 (4.9 – 7.6) | 0.596 |

Table E1: Absolute extravascular lung water index (EVLWI) values between treatment arms.

Placebo n=35 and vitamin D_3_ n=33 at preoperative and postoperative points; placebo n=32 and vitamin D_3_ n=31 at day 1 time point due to loss of PiCCO catheter. IQR – interquartile range; p-value represents Mann-Whitney test.

In a within group analysis EVLWI rose significantly preoperatively to postoperatively only in the placebo group (from median 6.3 IQR [5.3 – 7.7] to median 7.1 IQR [6.0 – 9.5] ml/kg, p=0.0002) whereas increases in the cholecalciferol treated group were not significant (from median 6.2 IQR [5.3 – 7.5] to 6.8 IQR [5.8 – 7.6] ml/kg, p=0.12) (Figure E1).

Figure E1: Box and whisker plot of absolute extravascular lung water index (EVLWI) values pre to postoperatively.

Placebo n=35, vitamin D_3_ n=33. Data presented as medians with Tukey’s distribution. *Circles* and *triangles* represent outlying values. P-values represent Wilcoxon matched-pairs signed rank test.

***Pulmonary vascular permeability index (PVPI)***

| PVPI  *median (IQR)* | Placebo  *n=35* | Cholecalciferol  *n=33* | p-value |
| --- | --- | --- | --- |
| Preoperative | 1.5 (1.3 – 1.7) | 1.7 (1.4 – 2.0) | 0.05 |
| Postoperative | 2.0 (1.6 – 2.1) | 1.8 (1.6 – 2.0) | 0.31 |
| Day 1 | 1.4 (1.1 – 1.6) | 1.4 (1.1 – 1.9) | 0.59 |

Table E2: Absolute pulmonary vascular permeability (PVPI) values between treatment arms.

Placebo n=35 and vitamin D_3_ n=33 at preoperative and postoperative points; placebo n=32 and vitamin D_3_ n=31 at day 1 time point due to loss of PiCCO_2_ catheter. IQR – interquartile range; p-value represents Mann-Whitney test.

PVPI also significantly increased pre to postoperatively in patients who received placebo (median 1.5 [IQR 1.3 – 1.7] to 2.0 [IQR 1.6 – 2.1], p=0.0002,) whereas patients who received cholecalciferol levels remained stable (median 1.7 [IQR 1.4 – 2.0] to 1.8 [IQR 1.6 – 2.1], p=0.36) (Figure E2).

Figure E2: Box and whisker plot of absolute values pulmonary vascular permeability index (PVPI) pre to postoperatively.

Placebo n=35, vitamin D_3_ n=33. Data presented as medians with Tukey’s distribution. *Circles* and *triangles* represent outlying values. P-values represent Wilcoxon matched-pairs signed rank test.

Figure E3: Box and whisker plots of fold change in A] EVLWI and B] PVPI in 25(OH)D deficient and sufficient patients.

25(OH)D_3_ <50nmol/L n=22; 25(OH)D_3_ >50nmol/L n=46. Data presented as medians with Tukey’s distribution. *Circles* and *triangles* represent outlying values. P-values represent Mann Whitney test.

***Safety of cholecalciferol supplementation***

Trial medication was well tolerated with 4 patients (2 placebo and 2 cholecalciferol) developing self-limiting gastrointestinal upset in the form of episodes of diarrhea up to 24 hours post drug administration. There were no episodes of hypercalcemia post drug administration. The frequency of serious adverse effects (SAEs) and adverse effects (AEs) was similar between the groups (online supplement Table E3). There were no reported SAEs related to the trial medication or suspected unexpected serious adverse events (SUSARs).

|  | Placebo (n=35) | Cholecalciferol (n=33) | P value |
| --- | --- | --- | --- |
| Hospital/Ventilator acquired Pneumonia | **12 (34%)** | **13 (39%)** | **0.66** |
| Reintubation | **7 (20%)** | **8 (24%)** | **0.67** |
| Mechanically Ventilated on day 1 post op | **4 (11%)** | **7 (21%)** | **0.27** |
| Any respiratory complication | **17 (49%)** | **17 (52%)** | **0.81** |
| Any infection | **15 (49%)** | **18 (55%)** | **0.34** |
| Any cardiac complication | **12 (34%)** | **6 (18%)** | **0.13** |
| Required inotropes in first 7 days post op | **18 (51%)** | **16 (48%)** | **0.81** |
| Renal replacement therapy | **0 (0%)** | **1 (3%)** | **0.49** |
| Returned to theatre | **1 (3%)** | **1(3%)** | **1.00** |
| Any surgical complication | **8 (23%)** | **12 (36%)** | **0.22** |

**Table E3: Adverse events summarized by treatment groups**

***Efficacy of cholecalciferol supplementation upon VD status perioperatively.***

In patients who received placebo, 25(OH)D but not 1,25(OH)_2_D or DBP levels decreased significantly between the day of randomization and the day of the operation (Table 3A). 1,25(OH)_2_D concentrations decreased significantly pre to postoperative day 3 in the placebo group but not in the cholecalciferol group and patients who received cholecalciferol had significantly higher concentrations of 1,25(OH)_2_D on day 3 post operatively (Table 3B).

**Clinical Outcomes**

There was no significant difference seen in PaO2:FiO2 ratio between the two groups postoperatively and at day 1. In total 8/68 (11.8%) patients developed ARDS with all of them occurring within 4 days of surgery with 5/8 (62.5%) within the first 24 hours postoperatively. There was no difference in ARDS rates between placebo and cholecalciferol treatment arms (placebo 4 [11.4%] of 35 compared with cholecalciferol 4 [12.1%] of 33; odds ratio 0.94; 95% CI 0.21 – 4.09). There was no difference seen in ventilator free and organ failure free days or survival (28 or 90 day)

***Organ dysfunction***

The majority of patients were not ventilated on any of the first 7 days post operatively and there was no significant difference between groups in ventilator free days in the first 7 days post op (median for both groups 7 days ventilator free, p=0.2799). 10 (29%) patients in the placebo group and 12 (36%) patients in the cholecalciferol group required ventilation at some point in the first 7 days after the operation (p=0.4924). There was no significant difference in SOFA score on day 1 post op (placebo median 3 (IQR 2-5), cholecalciferol median 4.0 (IQR 2-6), p=0.41). There was no significant difference in APACHE-II scores between groups (placebo mean 8.8 (SD 3.15), cholecalciferol mean 8.7 (SD 4.51), p=0.86). The median mortality predicted by the APACHE-II scores was 4.7% for placebo and 4.1% for cholecalciferol (p=0.42).

***Length of stay and Survival***

There was an increase in ICU length of stay in the cholecalciferol treated group (3 *vs.* 5 days, p=0.052) (Table E4). There was no significant difference between groups in length of hospital admission with median length of stay 13 days for both groups (p=0.74). A total of 4 patients who underwent esophagectomy died at 90 day follow-up (94.1% survived). There was no difference in 28 and 90 day survival between the 2 arms (Table E4)

Two patients died prior to hospital discharge. Both had received placebo. One patient died 24 days post randomization (preop total 25(OH)D=13.2nmol/L) and the other 73 days post randomization (preop 25(OH)D = 73.7nmol/L). The only patient who died within 28 days of randomization was the patient detailed above. 2 further patients (4 in total) died within 90 days of randomization. Both received cholecalciferol. One patient died 75 days post randomization (pre-op total 25(OH)D= 74.9nmol/L) and the other died 82 days post randomization (pre-op total 25(OH)D= 83.4nmol/L).

|  | Placebo  *n=35* | Cholecalciferol  *n=33* | p-value |
| --- | --- | --- | --- |
| ITU LOS, days  *Median (IQR)* | 4 (3 – 6) | 5 (3 – 10) | 0.052 |
| Hospital LOS, days  *Median (IQR)* | 13 (10 – 20) | 13 (11 – 23) | 0.739 |
| 30-day survival, n(%) | 34 (97.1) | 33 (100) | 1.0 |
| 90-day survival, n(%) | 33 (94.2) | 31 (93.9) | 1.0 |

**Table E4: Length of stay and survival.**

IQR, interquartile range; p-values represent Mann Whitney tests and Fisher’s exact test for categorical data.

***Markers of lung injury and systemic inflammation:***

There were no differences seen in perioperative markers of systemic inflammation (IL1β, IL-6, IL-8, IL-10, and TNF receptor 1) between the groups. In contrast, TNF receptor 2 (p=0.02) concentrations were higher at day 1 in cholecalciferol treated patients. Postoperative sRAGE concentrations were increased significantly in both groups suggesting Type 1 epithelial damage however there was no difference in sRAGE concentrations between the arms (Table E5).

|  | Time-point | Placebo | Cholecalciferol | p-value |
| --- | --- | --- | --- | --- |
| IL-1β  (pg/ml) | Pre-op  Post-op  Day 1  Day 3 | 7.8 (7.0 – 10.0)  9.3 (8.0 – 10.5)  8.5 (7.8 – 10.0)  9.3 (8.0-11.5) | 7.1 (6.0 – 8.0)  9.0 (7.5 – 10.5)  8.0 (7.0 – 10.0)  8.8 (7.6-12.5) | **0.007^*^**  0.78  0.23  0.64 |
| IL-6  (pg/ml) | Pre-op  Post-op  Day 1  Day 3 | 9.5 (8.5 – 11.3)  296.4 (188.5 – 488.5)  240.5 (133.8 – 444.4)  124.5 (68.1-329.3) | 9.8 (8.5 – 11.3)  546.3 (202.8 – 920)  249.0 (153.4 – 451.4)  162.0 (127.5-360.4) | 0.18  0.05  0.88  0.25 |
| IL-8  (pg/ml) | Pre-op  Post-op  Day 1  Day 3 | 48.9 (35.6 – 58.9)  87.1 (60.9 – 133.1)  85.3 (68.0 – 122.0)  92.6 (64.7-135.8) | 44.4 (36.7 – 53.1)  102.0 (74.3 – 156.0)  85.0 (68.5 – 151.6)  88.0 (71.5-172.8) | 0.22  0.17  0.97  0.39 |
| IL-10  (pg/ml) | Pre-op  Post-op  Day 1  Day 3 | 15.0 (14.0 – 16.5)  66.0 (31.5 – 106.5)  27.0 (21.0 – 36.4)  17.8 (16.0-22.3) | 15.0 (14.0 – 17.5)  43.0 (31.0 – 76.0)  26.5 (23.5 – 37.0)  20.0(16.3-24.5) | 0.39  0.31  0.67  0.35 |
| TNFα  (pg/ml) | Pre-op  Post-op  Day 1  Day 3 | 12.5 (11.0 – 13.5)  10.5 (9.5 – 11.5)  11.5 (10.4 – 13.6)  12.0 (10.6-13.5) | 12.5 (11.3 – 13.5)  11.0 (10.0 – 12.5)  12.0 (10.5 – 13.0)  13.0 (11.3-17.4) | 0.96  0.14  0.54  0.16 |
| TNFR-1  (pg/ml) | Pre-op  Post-op  Day 1  Day 3 | 392 (254 – 577)  806 (520 – 1302)  716 (468 – 1179)  1044 (518.3-1689) | 391 (300 – 596)  1181 (683 – 1443)  886 (606 – 1694)  958.3 (617.1-1531) | 0.49  0.09  0.05  0.85 |
| TNFR-2  (pg/ml) | Pre-op  Post-op  **Day 1**  Day 3 | 2365 (2003 – 3677)  3112 (2263 – 4879)  3807 (2403 – 4980)  5043 (3391-7505) | 2865 (2211 – 4340)  4148 (3125 – 5488)  5130 (3607 – 6864)  5000 (4134-7627) | 0.26  0.12  **0.02^*^**  0.34 |
| sRAGE  (pg/ml) | Pre-op  Post-op  Day 1  Day 3 | 43.0 (36.0 – 58.6)  51.0 (37.6 – 67.4)  35.3 (28.8 – 44.3)  36.5 (33.0-52.0) | 42.0 (33.9 – 51.3)  52.5 (39.0 – 91.0)  39.9 (30.6 – 64.1)  35.8 (28.1-45.5) | 0.57  0.76  0.13  0.33 |

**Table E5: Comparison of plasma markers of inflammation and epithelial damage.** Data presented as median (IQR)

**REFERENCES**

1. Parekh D, Dancer RC, Lax S, Cooper MS, Martineau AR, Fraser WD, Tucker O, Alderson D, Perkins GD, Gao-Smith F, Thickett DR. Vitamin D to prevent acute lung injury following oesophagectomy (VINDALOO): study protocol for a randomised placebo controlled trial. *Trials* 2013; 14: 100.

2. Perkins GD, McAuley DF, Thickett DR, Gao F. The beta-agonist lung injury trial (BALTI): a randomized placebo-controlled clinical trial. *Am J Respir Crit Care Med* 2006; 173: 281-287.

3. Craig TR, Duffy MJ, Shyamsundar M, McDowell C, O'Kane CM, Elborn JS, McAuley DF. A randomized clinical trial of hydroxymethylglutaryl- coenzyme a reductase inhibition for acute lung injury (The HARP Study). *American journal of respiratory and critical care medicine* 2011; 183: 620-626.

4. Craig TR, Duffy MJ, Shyamsundar M, McDowell C, McLaughlin B, Elborn JS, McAuley DF. Extravascular lung water indexed to predicted body weight is a novel predictor of intensive care unit mortality in patients with acute lung injury. *Critical care medicine* 2010; 38: 114-120.

5. Kushimoto S, Taira Y, Kitazawa Y, Okuchi K, Sakamoto T, Ishikura H, Endo T, Yamanouchi S, Tagami T, Yamaguchi J, Yoshikawa K, Sugita M, Kase Y, Kanemura T, Takahashi H, Kuroki Y, Izumino H, Rinka H, Seo R, Takatori M, Kaneko T, Nakamura T, Irahara T, Saito N, Watanabe A, Group PPES. The clinical usefulness of extravascular lung water and pulmonary vascular permeability index to diagnose and characterize pulmonary edema: a prospective multicenter study on the quantitative differential diagnostic definition for acute lung injury/acute respiratory distress syndrome. *Crit Care* 2012; 16: R232.

6. Perkins GD, Nathani N, McAuley DF, Gao F, Thickett DR. In vitro and in vivo effects of salbutamol on neutrophil function in acute lung injury. *Thorax* 2007; 62: 36-42.

7. Owens DJ, Webber D, Impey SG, Tang J, Donovan TF, Fraser WD, Morton JP, Close GL. Vitamin D supplementation does not improve human skeletal muscle contractile properties in insufficient young males. *Eur J Appl Physiol* 2014; 114: 1309-1320.
